# A study on the relationship between learning burnout and quality of life among primary and secondary school students during an infectious disease epidemic: the mediating roles of depression and family health

**DOI:** 10.1186/s12888-025-07353-7

**Published:** 2025-09-26

**Authors:** Xinyuan Wei, Lixia Liang, Yiqing He, Na Shao, Lu Yang, Xiaojie Yuan, Huiyun Yang, Hui Yang

**Affiliations:** 1 Department of Urology, West China Hospital, Sichuan University, Chengdu, 610041 China; 2 Department of Otolaryngology and Head Surgery, The Second Affiliated Hospital of Xi’an Jiaotong University, Xi’an, 710004 China; 3 Department of Oncology Surgery, The Second Affiliated Hospital of Xi’an Jiaotong University, Xi’an, 710004 China; 4 Department of Epidemiology, School of Public Health, Air Force Medical University, Xi’an, Xi’an, 710000 People’s Republic of China; 5 Nursing Department, The Second Affiliated Hospital of Xi’an Jiaotong University, Xi’an, 710004 China

**Keywords:** Public health safety incidents, Primary and secondary school students, Learning burnout, Family health, Depression, Quality of life

## Abstract

**Background:**

To investigate the mediating roles of depressed mood and family health in the relationship between learning burnout and quality of life (QOL) among primary and secondary school students during public health emergencies.

**Methods:**

The study was conducted in Xi’an City, China, from 15 to 30 January 2022, targeting students from urban and rural areas within the epidemic prevention and control zone. A total of 20,152 questionnaires were initially collected, with 809 invalid responses excluded, resulting in a final sample of 19,343 valid questionnaires (response rate of 95.99%) from primary and secondary school students. Regression analysis and mediation effect analysis were employed to assess the relationships among learning burnout, depression, family health, and QOL.

**Results:**

Learning burnout was negatively associated with QOL, and the association was potentially mediated through depression and family health. Depression was inversely related to QOL, with family health potentially acting as a mediator in this relationship, exhibiting a healthy family was correlated with higher QOL (depression→family health: -0.87, family health→QOL: 0.66), which was inversely related to the negative effects of depression (depression→QOL: -0.11).

**Conclusions:**

Depression and family health were identified as potential mediators in the association between learning burnout and QOL among these students, highlighting the crucial importance of alleviating depression and enhancing family health in efforts to improve their overall QOL.

**Supplementary Information:**

The online version contains supplementary material available at 10.1186/s12888-025-07353-7.

## Background

A sudden public health event is defined as a major infectious disease outbreak, an unexplained mass disease or another event that significantly impacts the population’s health [1]. In late December 2021, the city of Xi’an experienced a severe outbreak of COVID-19, potentially resulting in psychological issues, which negatively affect interpersonal relationships and the economy, ultimately diminishing the public’s quality of life (QOL) in the long run [2, 3]. Learning burnout refers to the emotional exhaustion, dehumanization, and low personal fulfillment that students experience due to prolonged learning pressure [4, 5]. Widespread online learning during the pandemic has impacted students’ lives and mental health, resulting in an increasing prominence of learning burnout, subsequently leading to a decline in academic grades and a reduction in sleep quality [6].

Family health serves as a critical role in psychological well-being. Robust family health buffers stress by enhancing emotional regulation and providing instrumental support, whereas poor family health correlates with increased psychological vulnerability [7]. For graduate students, family functionality, social support, and effective coping mechanisms reduce academic burnout [8], while a 24.2% incidence of depression was reported among college students [9]. In the familial milieu, the capacity of parental emotion regulation exerts a significant positive influence on children’s emotion regulation [10]. A positive family atmosphere and a high level of individuation are associated with lower levels of depressive symptoms. Moreover, these factors have certain diagnostic value for adolescent depression [11]. The Ecological Systems Theory provides a theoretical framework for understanding the interplay among these variables, emphasizing the dynamic interrelations between environmental and individual factors. Family adaptability directly influences adolescents’ mental health and reduces the risk of depression by providing emotional support and fostering effective communication patterns. Simultaneously, family adaptability indirectly affects depressive symptoms by enhancing perceived social support and self-efficacy [12].

Family health and depression may play important roles between students’ learning burnout and QOL. Data on medical students’ mental health indicate that the emotional exhaustion and cynicism dimensions of learning burnout are positively correlated with depression. Depression, in turn, negatively impacts health status. However, academic self-efficacy can indirectly improve health status by reducing the risk of depression [13]. In the online learning environment during the COVID-19 pandemic, stress significantly exacerbated academic burnout among medical students, while psychological resilience directly buffered its negative effects. Social support, though not directly influencing burnout, played an indirect protective role by enhancing psychological resilience [14]. It is evident that family support, as a crucial component of social support, plays a pivotal role in this process.

Existing studies predominantly focus on college students, leaving a critical void in understanding learning burnout and QOL among primary and secondary school students during public health crises, though some studies involved environment, ability, relation, emotion, and learning burnout on adolescents [15–17].

Therefore, This study aims to assess the current state of family health levels, depressive symptoms, learning burnout and QOL among primary and secondary school students during this public health and safety incident. It also seeks to analyze the relationships among these factors to provide insights for improving family dynamics and developing national mental health strategies for primary and secondary school students during public safety incidents.

## Methods

### Research objectives

This study employed a convenience sampling method to select primary and secondary school students from urban and rural areas within the epidemic prevention and control zone of Xi’an City, from 15 to 30 January 2022, as the study population. The inclusion criteria were (1) primary and secondary school students residing in the 10 districts and 3 counties of Xi’an City during the survey period, (2) aged 6 to 18 years old, (3) capable of using mobile phones independently or using mobile phones to answer questions with parental assistance, and (4) of normal cognitive function and sound mental health, without major diseases affecting motor or speech functions. The exclusion criteria included (1) a history of mental illness and consciousness disorders and (2) participation in other clinical trials. We initially excluded students with confirmed diagnosis of mental health disorders, as well as those suspected of having such conditions, through the school. Subsequently, an electronic questionnaire Link was distributed to the selected students and their parents. To ensure the representativeness and diversity of the sample, we also included students from rural areas, where the population is relatively sparse and the Pandemic control measures were less stringent. Ultimately, a total of 20,152 questionnaires were collected. After screening and excluding 809 invalid responses, 19,343 valid questionnaires were included in the analysis, representing an effective response rate of 95.99%. This study was approved by the ethics committee of our hospital (No. 2022001). The authors assert that all procedures contributing to this work comply with the ethical standards of the relevant national and institutional committees on Human experimentation and with the Helsinki Declaration of 1975, as revised in 2008. Informed consent was required from the legal guardians of the primary and secondary school students, and when possible, consent was obtained directly from the students able to understand and agree to participate in the study.

### Research tools

#### General information questionnaire for research participants

The classic and authoritative four questionnaires recognized for their strong reliability and validity were adapted to gather data on age, gender, ethnicity, educational stage, place of residence, epidemic control status in the place of residence and teachers’ instruction methods.

#### Short form of the family health scale

The Family Health Scale–Short Form (FHS–SF), compiled by Crandall et al. [18], comprises four dimensions: family social and emotional health processes, family healthy Lifestyle, family Health resources and family external social support, totalling 10 items. It employs a 5-point Likert scale, with 1 representing ‘strongly disagree’, 2 ‘somewhat disagree’, 3 ‘neither agree nor disagree’, 4 ‘somewhat agree’ and 5 ‘strongly agree’. The cumulative score is used to assess family health: a total score < 25% (12.5 points) indicates poor family health; a total score between 25% and 75% (12.5 points to 37.5 points) indicates moderate family health, and a score > 75% (more than 37.5 points) indicates good family health. Wang et al. [19] applied this scale in a survey of 8,912 residents across 120 cities in China, confirming its good reliability and validity. Based on the current sample data, the Cronbach’s alpha value of this scale is 0.675, indicating that it has high reliability.

#### Center for epidemiological studies depression scale

The Center for Epidemiological Studies Depression Scale (CES-D) was developed by Radloff at the National Institute of Mental Health [20] and is used in epidemiological surveys to screen participants for symptoms of depression, facilitating further diagnostic testing. It emphasises the individual’s emotional experience more than other depression self-rating depression scales. The CES-D includes 20 items, each assessing a symptom of depression, and it has been validated for clinical use with good reliability and validity. The scoring method is based on the frequency of symptoms over the past week: 0 for ‘none or almost none’ (less than 1 day), 1 for ‘rarely’ (1–2 days), 2 for ‘often’ (3–4 days) and 3 for ‘almost always’ (5–7 days). A total score below 10 indicates no symptoms of depression; 10 to 15 suggests possible symptoms of depression, and a score of 20 or more indicates definite symptoms of depression. Based on the current sample data, the Cronbach’s alpha value of this scale is 0.912, indicating that it has high reliability.

#### Adolescent learning burnout scale

This scale, developed by Wu et al. in 2007 [21], assesses learning burnout in adolescents. It comprises three dimensions: physical and mental exhaustion, learning detachment and low achievement (with a total of 16 items). A 5-point Likert scale was employed, with scores from 1 to 5 representing various degrees of agreement ranging from ‘very much agree’ to ‘strongly disagree’. The total score is the sum of the scores of the 16 items, with higher scores indicating more severe burnout conditions among students. Based on the current sample data, the Cronbach’s alpha value of this scale is 0.901, indicating that it has high reliability.

#### QOL scale for children and adolescents

The QOL Scale for Children and Adolescents (QLSCA) is a self-rating scale designed for children and adolescents to evaluate their multidimensional QOL, focusing on their learning life [22]. It includes 49 items across 13 dimensions (self-satisfaction, teacher–student relationship, somatic feeling, peer relationship, parent–child relationship, motor ability, learning ability and attitude, self-concept, negative emotion, attitude towards homework, activity accessibility, convenience of life and others), grouped into four factors (psychosocial functioning, physiological and psychological well-being, living environment and satisfaction with QOL). A 4-point Likert scale was used, with 1 signifying ‘never’, 2 ‘seldom’, 3 ‘often’ and 4 ‘always’. Higher scores denote a better QOL. Based on the current sample data, the Cronbach’s alpha value of this scale is 0.951, indicating that it has high reliability.

### Data collection methods

Data collection was conducted using the Questionnaire Star online survey platform. Clear instructions were provided before filling out the questionnaire. Informed consent was obtained through a multiple-choice question; participants who chose not to agree were not allowed to proceed with the questionnaire. Students completed the questionnaire independently or with parental assistance, taking approximately 8–10 min to complete. The system included a self-checking function to ensure that all items were completed before submission. Regarding the informed consent process for minor participants, we informed and obtained consent from parents or legal guardians through online parent groups. Specifically, we provided detailed information to the parents regarding the purpose, content, potential risks, and measures to protect the rights of minors in our study, ensuring that they fully understood and agreed to their children’s participation. After obtaining parental consent, parents and children completed the questionnaire together.

### Outcomes

The initial score (X) was obtained, and the corresponding national normative mean score (M) and standard deviation (SD) were determined based on each participant’s place of residence (urban or rural) and age. To facilitate a more meaningful comparison with national norms and to standardize the data across participants from different age groups and urban-rural backgrounds, T-scores were employed in the analysis. By converting raw scores into standardized scores, T-scores offer a more intuitive representation of an individual’s relative standing within the population, thereby enabling more robust cross-group comparisons. This approach ensures that demographic variations do not bias the results, allowing for a more precise and valid interpretation of the data. The standardized T-score was calculated using the formula $\:T=50+(X-M)/SD\ast\:10$, and was used to represent the QOL as a continuous variable, allowing for more precise comparisons across different groups.

### Data analysis

Variables are presented as means with their corresponding standard deviation (SDs), as medians with quartiles or as simple numbers with percentages. The Student’s T test, Wilcoxon’s test, standard chi-square test and Fisher’s Exact test were used to compare the participants’ characteristics between males and females. QOL score was described and compared among participants of different education level, ethnicity, place, control area, isolation method, teaching method, learning burnout, depression and family health status.

Pearson’s correlation was employed to test the correlation between item scores and total scores. Univariate and multivariate-adjusted linear regression models with stepwise selection method were utilised to estimate the association between factors and QOL. β and standard error (SE) was calculated. Restricted cubic splines with three knots (p5, p50 and p95) were further utilised to assess the linearity between learning burnout, depression, family health and QOL after adjusting confounding variables. To estimate the mediation effect of depressive symptom and family health, the ‘mediation’ R package was used to estimate the direct and indirect effect of learning burnout, depression and family health, respectively. Four equation was built and β, its 95% confidence interval (CI) as well as mediation proportion was calculated. All tests were two-sided, with the threshold for statistical significance set at *p* < 0.05. All analyses were performed using SAS software version 9.4 and R software version 2.13.2.

## Results

### Sample characteristics

A total of 19,343 primary and secondary school students were included in the questionnaire for this study. Analysis of the categorical variables with gender indicated a statistically significant difference in the learning burnout, depression, family health and QOL scores when compared by gender (*p* < 0.05), with the results presented in Table 1.

**Table 1:** Baseline characteristics of participants

	All	Male	Female	*p*
Number	19,343	9644 (49.8)	9699 (50.2)	
Age	11.6 ± 3.0	11.6 ± 3.0	11.6 ± 2.9	0.189
Education				0.118
Primary	8880 (55.3)	4483 (56.1)	4397 (54.6)	
Junior	5015 (31.2)	2471 (30.9)	2544 (31.6)	
Senior	2159 (13.5)	1042 (13.0)	1117 (13.8)	
Ethnicity				0.529
Han	15,797 (98.4)	7863 (98.3)	7934 (98.5)	
Hui	257 (1.6)	133 (1.7)	124 (1.5)	
Place				0.774
Rural	3402 (21.2)	1687 (21.1)	1715 (21.3)	
Urban	12,652 (78.8)	6309 (78.9)	6343 (78.7)	
Control and prevention				0.259
Closed area	1346 (8.4)	669 (8.4)	677 (8.4)	
Controlled area	13,772 (85.8)	6885 (86.1)	6887 (85.5)	
Precautionary area	936 (5.8)	442 (5.5)	494 (6.1)	
Isolation method				0.998
Home quarantine	6622 (41.2)	3300 (41.2)	3322 (41.2)	
Hotel isolation	44 (0.3)	22 (0.3)	22 (0.3)	
Not isolated	9388 (58.5)	4674 (58.5)	4714 (58.5)	
Teaching methods				0.224
Online teaching	15,911 (99.1)	7932 (99.2)	7979 (99.0)	
Face-to-face sessions	143 (0.9)	64 (0.8)	79 (1.0)	
Learning burnout	43.0 ± 6.3	43.2 ± 6.4	42.9 ± 6.1	0.001
Depression	18.7 ± 7.8	18.3 ± 7.5	19.1 ± 8.0	< 0.001
Family health	42.9 ± 4.6	42.7 ± 4.6	43.0 ± 4.6	0.004
Quality of life	55.8 ± 13.0	56.8 ± 12.7	54.8 ± 13.2	< 0.001

The QOL scores of primary and secondary school students during public health events were statistically different across subgroups of education, region and prevention and control measures (*p* < 0.05), as shown in Table 2.

**Table 2:** Quality of life among subgroups with different characteristics

	Quality of life (mean ± SD)	*p*
Education		< 0.001
Primary	57.3 ± 11.8	
Junior	53.6 ± 14.4	
Senior	54.5 ± 13.6	
Ethnicity		0.8301
Han	55.8 ± 13.0	
Hui	55.6 ± 13.3	
Place		< 0.001
Rural	56.9 ± 13.8	
Urban	55.5 ± 12.8	
Control and prevention		< 0.001
Closed area	54.0 ± 13.9	
Controlled area	55.9 ± 12.9	
Precautionary area	56.4 ± 13.3	
Isolation method		< 0.001
Home quarantine	54.8 ± 13.4	
Hotel isolation	51.2 ± 14.6	
Not isolated	56.5 ± 12.7	
Teaching methods		0.0491
Online teaching	55.8 ± 13.0	
Face-to-face sessions	53.7 ± 13.3	
Learning burnout		< 0.001
Yes	54.8 ± 13.9	
No	57.0 ± 11.7	
Depression		< 0.001
Yes	47.6 ± 11.7	
No	61.3 ± 10.7	
Family health		< 0.001
Yes	57.1 ± 12.3	
No	43.6 ± 12.8	

### Linear association between learning burnout, depression, family health and QOL

**Fig. 1:**
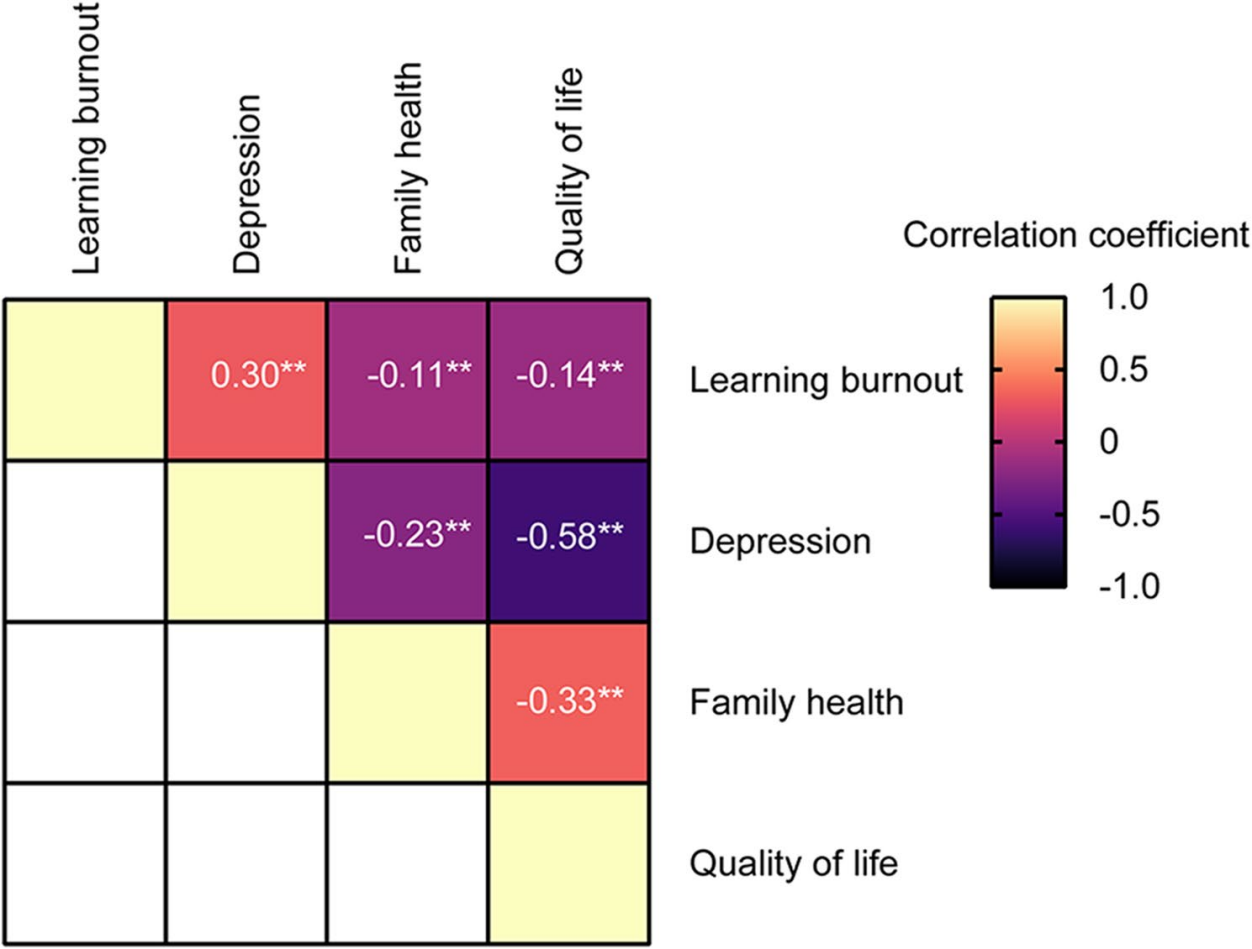
Correlation analysis of learning burnout, depression, family health and quality of life. ** represents *p* < 0.01

The correlation analysis highlighted significant associations: learning burnout showed a moderate positive correlation with depression (*r* = 0.30, *p* < 0.01) and a negative correlation with QOL (*r* = −0.14, *p* < 0.01). Depression had a strong negative impact on QOL (*r* = −0.58, *p* < 0.01). Family health was negatively correlated with both depression (*r* = −0.23, *p* < 0.01) and QOL (*r* = −0.33, *p* < 0.01) (Fig. 1), suggesting that the relationships between the variables are complex. Learning burnout tends to exacerbate depression, negatively affecting family health and QOL. Depression has a significant detrimental effect on QOL. The negative correlation between family health and QOL may be influenced by other factors not included, such as economic conditions.

### Linear regression model

Linear regression analysis identified age, gender, education level, urban residency, isolation methods, face-to-face sessions, learning burnout, depression and family health as independent risk factors for QOL among primary and secondary school students, as shown in Table 3. In the univariate analysis, learning burnout (β = −0.305, *p* < 0.001) and depression (β = −0.982, *p* < 0.001) had a significant negative impact on QOL, while family health (β = 1.049, *p* < 0.001) had a significant positive impact. In the multivariate analysis, after controlling for variables such as age, gender, and education level, the direct effect of learning burnout on QOL became positive (β = 0.129, *p* < 0.001). Depression (β = −0.902, *p* < 0.001) continued to have a significant negative effect on QOL, and family health (β = 0.660, *p* < 0.001) had a significant positive impact. This suggests that learning burnout may indirectly affect QOL through mediating variables, while the independent effects of depression and family health remain stable.

**Table 3:** Linear regression model

Variables	Univariate			Multivariate		
	β	SE	*p*	β	SE	*p*
Age	−0.27243	0.03446	< 0.001	0.52723	0.04511	< 0.001
Male	2.016	0.20464	< 0.001	1.28218	0.15843	< 0.001
Education			< 0.001			< 0.001
Primary	1			1		
Junior	−3.74087	0.22762		−3.38964	0.25276	
Senior	−2.81795	0.30922		−2.12451	0.36871	
Hui	−0.17546	0.81771	0.8031			
Urban	−1.45997	0.25087	< 0.001	−2.27294	0.19629	< 0.001
Control and prevention			< 0.001			
Closed area	1					
Controlled area	1.97098	0.37101				
Precautionary area	2.47678	0.55292				
Isolation method			< 0.001			< 0.001
Home quarantine	1			1		
Hotel isolation	−3.64016	1.96282		−2.18575	1.51269	
Not isolated	1.63679	0.20825		0.64649	0.16228	
Face-to-face sessions	−2.14987	1.09216	0.049			
Learning burnout	−0.30452	0.0163	< 0.001	0.12946	0.01348	< 0.001
Depression	−0.9822	0.01072	< 0.001	−0.90216	0.01133	< 0.001
Family health	1.0492	0.02082	< 0.001	0.66019	0.01816	< 0.001

### Analysis of chain mediation effects

**Fig. 2:**
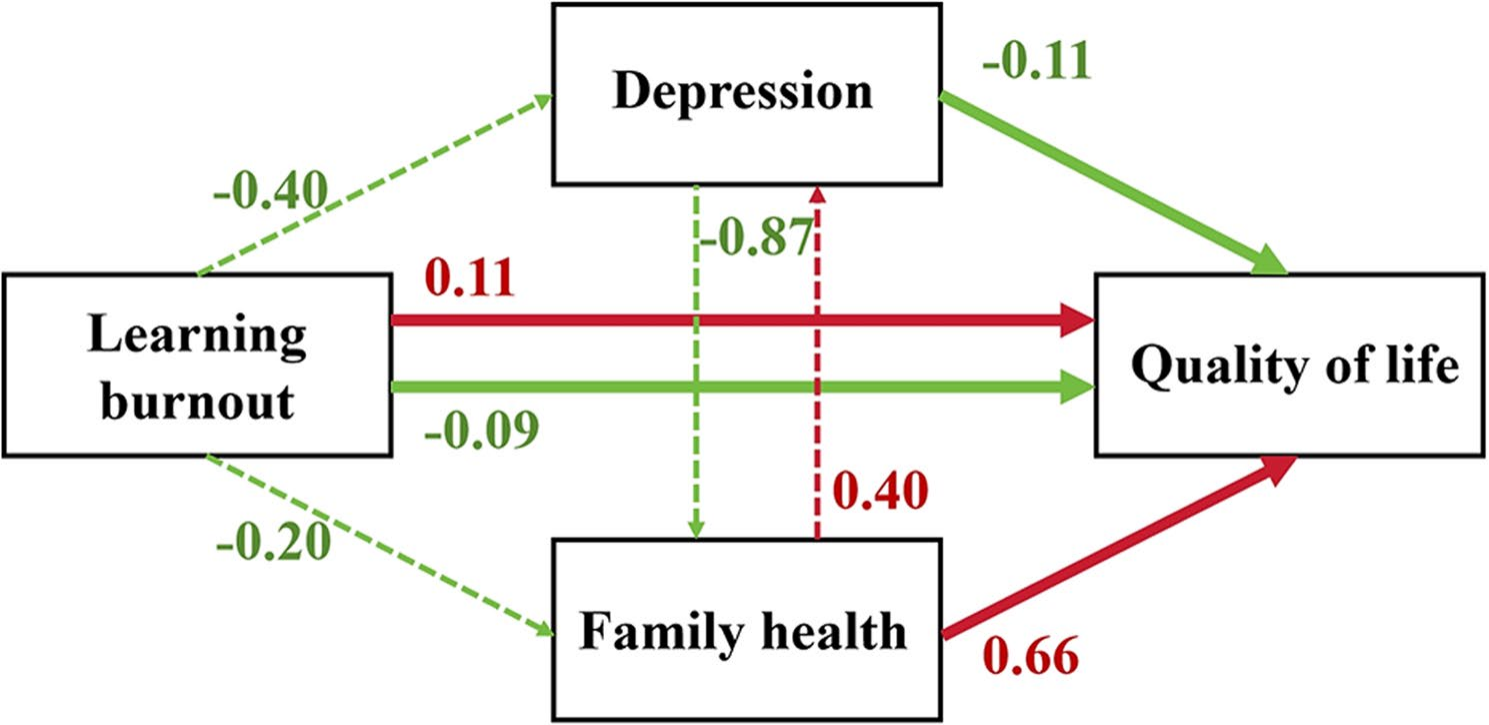
Depression symptom mediation models of the relationships between learning burnout, depression, family health and quality of life. The dotted lines represent indirect effects; the solid lines denote direct effects; the red lines indicate positive relationships; and the green lines represent negative relationships. All equations included age, gender, education, district, isolation method and teaching method as covariates

Learning burnout was significantly associated with lower QOL through indirect pathways involving depression and family health. Learning burnout was associated with a negative indirect effect on QOL via depression (indirect effect = −0.40, 95% CI [−0.43, −0.38]), accounting for 139% of the total effect. Additionally, learning burnout was associated with reduced QOL through the partial mediation of family health (indirect effect = −0.20, 95% CI [−0.23, −0.17]), representing 31% of the total effect. Conversely, depression also negatively impacted QOL through family health (indirect effect = −0.87, 95% CI [−0.89, −0.84]), with 15% of this relationship mediated by family health. These findings highlight that depression and family health are critical mediating factors in the association between learning burnout and QOL (Additional file 1 and Fig. 2).

## Discussion

The study showed correlations among learning burnout, depression, family health, and QOL, with a particularly moderate passive correlation observed between depression and QOL. Age, gender, educational level, urban residence, methods of isolation, face-to-face meetings, learning burnout, depression, and family health are independent risk factors for the QOL in primary and secondary school students. Compared to direct effects, learning burnout exhibited a stronger association with QOL through the mediating effects of depression and family health.

Learning burnout is a persistent negative psychological state related to learning, manifested by issues such as decreased concentration, depression and anxiety, and a decline in self-confidence [23]. The three dimensions of learning burnout, namely emotional exhaustion, depersonalization, and reduced academic efficacy, are notably negatively correlated with QOL, with emotional exhaustion exerting an especially pronounced effect [24]. The pandemic accelerated the popularity of online education, but it also triggered academic anxiety among college students due to issues such as unstable internet connections, insufficient preparation of teachers for online instruction, and difficulties in students’ understanding, which directly impact an individual’s QOL [6]. Life satisfaction is a crucial indicator for assessing QOL, and surveys targeting medical graduate students in China confirmed a significant negative correlation between learning burnout and life satisfaction [25]. Our study confirms this viewpoint and finds that 31% of the relationship is mediated by family health. Increased learning burnout was associated with lower family health, and lower family health was further linked to reduced QOL of secondary school students. A study found that following the third wave of the COVID-19 pandemic, the health-related QOL of primary and secondary school students was closely related to their family’s educational level. Families with lower education risked a reduced health-related QOL for children [26]. A decrease in family income during a public health event can also adversely affect the emotional well-being of family members [27]. Research on family stress modeling suggests that problems or coping methods within families under different economic pressures directly impact the emotions of minor children over time [28]. Therefore, when facing their children’s learning burnout, parents should provide more support, companionship, trust and encouragement.

In the context of public health and safety incidents, the level of family health among primary and secondary school students serves as an indicator of family members’ attitudes towards online learning, fostering positive interactions within the family. This environment enables parents to influence their children’s behaviours through leading by example and engaging in healthy emotional exchanges. Consequently, primary and secondary school students are more inclined to embrace learning, take the initiative in their studies, boldly engage in learning exploration, adapt more swiftly to online teaching methods and are less prone to experiences of disengagement, boredom and other learning burnout-related behaviours. The presence and support of family members during challenging times contribute significantly to the development of a strong parent–child relationship. This is crucial in helping children form a positive life outlook, establish correct values and develop a well-rounded personality, which is vital for enhancing their QOL in both the short and long term.

Recognising both the immediate and long-term effects, we should pay attention to the impact of family health on the learning burnout experienced by primary and secondary school students. A positive family atmosphere can provide children with a more expansive developmental space, making them feel genuinely supported and valued by their families. This support is instrumental in mitigating burnout and other negative psychological issues. During periods spent at home, fostering deep familial bonds enables children and their families to establish deep affection and the necessary resilience to effectively navigate life’s challenges, resulting in an improved QOL.

Learning burnout has a slightly direct positive relationship with QOL, which may be associated with the guilt resulting from learning burnout. Guilt also serves as a motivator for students to actively seek knowledge, cultivate beneficial learning habits, and ultimately enhance their QOL. A study also demonstrated that online instruction could stimulate student motivation, which contributes to academic success [6]. This phenomenon mirrors the relationship between post-traumatic stress symptoms and growth observed in adolescents after earthquakes [29]. We also found that depression brought about by learning burnout markedly reduces the QOL, which is consistent with previous studies [30]. Depression and burnout share similarities, which may complicate differentiation for primary and secondary school students [31]. This confusion may be associated with higher susceptibility to depression of students with learning burnout and contributes to a rise in their depressive symptoms. Increased depressive symptoms, along with the absence of school life, reduced outdoor activity, decreased peer interaction, etc., collectively diminish the QOL for primary and secondary school students. Consequently, when facing public health and safety incidents, a combined effort from schools and families is essential to conquer the burnout and depression of students. Schools could establish psychological profiles for these categories of students and introduce mental health programs and counseling services to aid students, including teaching them to manage their negative emotions and to adjust or motivate themselves appropriately. Families can strengthen their relationships through developing a robust family support system to prevent declines in students’ QOL.

Family health was positively associated with QOL, with 38% of this association mediated through depression. Family health alleviates depression in secondary school students, which in turn enhances their QOL. Within the same family environment, the lower the depression levels among primary and secondary school students, the better their QOL, aligning with the findings of Zhu et al. [32]. Furthermore, isolation measures during the COVID-19 pandemic have been shown to significantly increase anxiety and depressive symptoms among university students [33], suggesting that similar effects may be observed in younger students. In addition, a study among pre-university students found that certain parenting styles are associated with a higher prevalence of mental disorders in adolescents [34], further emphasizing the need for robust family support systems to mitigate these adverse impacts. During public health events, primary and secondary school students experience heightened feelings of panic, anxiety, depression and other emotions. The extended period of home study and life, along with restricted communication with their classmates, prevents students from expressing their negative emotions, highlighting the critical role of the family in the situation. Families could proactively manage risk in daily life and devise corresponding emergency plans to assist children in overcoming negative feelings, enabling children to handle emergencies smoothly and ensuring a positive life for them.

Students of this age are at a critical stage of growth and development, experiencing obvious physical and psychological changes. This period is the peak time for the emergence of depression and other adverse emotions that can persist into adulthood [35]. The gap between reality and the instinctive belief that the family is a refuge further exacerbates depression. Learning burnout is also exhibiting a trend of a younger onset [23]. Therefore, under public health events, it is vital to pay closer attention to these children and to actively explore the integration of school–family and teacher-parent educational models, especially for children with absent parents. Tailored interventions should be developed to meet the individual needs, aiming to help them mitigate detrimental mental effects and lessen the decline in their QOL.

Primary and secondary school students’ inaccurate self-perception and overestimation can lead to depression; similarly, parents constantly sharing their own hardships can also trigger depression in these students. The instinctive belief among primary and secondary school students that the family is a refuge for seeking help and expressing emotions can further exacerbate depression, especially if their emotional expressions to their parents do not yield the expected outcomes. Therefore, it is crucial to provide primary and secondary school students with correct value orientations, enabling them to accurately understand and assess themselves. Establishing an online channel for these students to address their unmet needs could also be beneficial. Parents should strive to create a supportive and united family atmosphere and respect their children’s perspectives to mitigate the onset of depression in their children and its impact on their QOL.

Depression is negatively associated with QOL, with 15% of this relationship mediated indirectly through family health. Depression undermines the family health of secondary school students, which in turn negatively affects their QOL. Family health significantly influences the mental health of primary and secondary school students. A high level of family health can foster the mental health development of these students, especially in the face of public health crises, where communication and mutual support among family members are crucial for managing stress. Conversely, students from families with low levels of family health often experience a lack of communication with family members when facing educational and interpersonal challenges, receiving little to no support from their families. A study has shown that the probability of children with one or two absent parents suffering from depression is 29% [36]. Changes in family structure have emerged as one of the main stressors in these students’ lives. A relative lack of parental affection can lead to children feeling isolated. This deficiency in parental affection predisposes children to develop withdrawal and feelings of inferiority, creating barriers in their interactions with teachers and classmates, thereby fostering more psychological problems, such as depression, which in turn diminishes the QOL for primary and secondary school students. Moreover, the lack of a stable and supportive family environment can significantly hinder a child’s ability to cope with stress and adversity, making them more susceptible to mental health issues [37].

Students of this age are at a critical stage of growth and development, experiencing significant physical and psychological changes. This period is the peak time for the emergence of depression and other adverse emotions that can persist into adulthood [35]. Therefore, in the context of public health events, it is vital to pay closer attention to the trends of depression among primary and secondary school students under different family health conditions. There is a need to actively explore the integration of school–family and teacher–parent educational models, especially for primary and secondary school students with absent parents. Tailored interventions should be developed, customising education to meet the individual needs of each student. This approach aims to help these students mitigate their depression, reduce the detrimental effects of a poor family environment on their mental health and lessen the decline in their QOL. Only by addressing these issues can we hope to alleviate the depression experienced by these students, reduce the negative impact of unfavourable family environments on their mental health and improve their QOL. To effectively address student mental health during public health crises, coordinated efforts across multiple stakeholders are essential. Schools should implement mental health programs and training for educators to identify and manage learning burnout and depression. Families must enhance emotional support through open communication and education on effective parenting strategies. Policymakers need to allocate resources for mental health services and develop guidelines to create supportive learning environments.

For the first time, we have revealed the significant mediating roles of both depression and family health in the relationship between learning burnout and QOL under a major public health crisis and demonstrated the significant impact of these factors on primary and secondary school students. In addition to these factors, other variables may also play a significant role in shaping students’ perceptions and expressions of burnout, anxiety, and depression. The mental and cognitive states of individuals and their families and spiritual experience are crucial in the context of psychological well-being [38, 39]. Furthermore, during the COVID-19 pandemic, students’ motivation and the modern online teaching technologies have had a profound impact on mental health [40]. These variables warrant further investigation in future research. However, the study has several limitations. The cross-sectional design precludes the establishment of causality, as it cannot determine the temporal order of variables or distinguish whether learning burnout precedes depression and QOL decline or vice versa. The sample is confined to primary and secondary school students in Xi’an, which limits the generalizability of the findings. Additionally, the reliance on self-reported questionnaires may introduce subjective bias. Additionally, potential confounders including socioeconomic status or family income were not fully explored, potentially affecting the accuracy of the mediational models. The investigation focused solely on depression and family health as mediators, neglecting other theoretically relevant constructs such as self-efficacy and social support networks that may influence the relationship between learning burnout and QOL. Future research could adopt longitudinal designs, recruit samples from multiple regions, and incorporate biological markers to reduce self-report bias. Additionally, testing other potential mediators (self-efficacy and social support) would provide more robust evidence.

## Conclusions

Learning burnout, depression, family health, and QOL are markedly correlated each other in primary and secondary school students under a major public health crisis, with depression showing a moderate negative correlation with QOL. Age, gender, educational level, urban residence, methods of isolation, face-to-face meetings, learning burnout, depression, and family health are independent risk factors of QOL. The association between learning burnout and QOL was more strongly mediated by depression and family health.

## Supplementary Information

Supplementary Material 1.

Supplementary Material 2.

Supplementary Material 3.

## Data Availability

The datasets used and/or analyzed during the current study are available from the corresponding author on reasonable request.

## Abbreviations

CES-D: Center for Epidemiological Studies Depression Scale
FHS–SF: Family Health Scale–Short Form
QLSCA: Quality of Life Scale for Children and Adolescents
SD: Standard deviation
