# Supplementary material for: A study on the relationship between learning burnout and quality of life among primary and secondary school students during an infectious disease epidemic: the mediating roles of depression and family health
Source: BMC Psychiatry. 2025 Sep 26;25:875. doi: 10.1186/s12888-025-07353-7 (PMC12465402; doi:10.1186/s12888-025-07353-7)
Supplement: Supplementary file 1 — Supplementary Material 1. [file 12888_2025_7353_MOESM1_ESM.docx]

Additional file 1 Analysis of chain mediation effects*

| Mediation effect pathway | Estimate | 95% CI | Mediation proportion |
| --- | --- | --- | --- |
| Equation 1 |  |  |  |
| Family health—Quality of life | 0.66 | 0.63, 0.69 |  |
| Family health—Depression—Quality of life | 0.40 | 0.38, 0.43 | 38% |
| Equation 2 |  |  |  |
| Depression—Quality of life | -0.11 | -0.12, -0.10 |  |
| Depression—Family health—Quality of life | -0.87 | -0.89, -0.84 | 15% |
| Equation 3 |  |  |  |
| Learning burnout—Quality of life | 0.11 | -0.08, 0.14 |  |
| Learning burnout—Depression—Quality of life | -0.40 | -0.43, -0.38 | 139% |
| Equation 4 |  |  |  |
| Learning burnout—Quality of life | -0.09 | -0.10, -0.08 |  |
| Learning burnout—Family health—Quality of life | -0.20 | -0.23, -0.17 | 31% |

* Adjusting for age, gender, education, district, isolation method and teaching methods
