# Supplementary material for: A study on the relationship between learning burnout and quality of life among primary and secondary school students during an infectious disease epidemic: the mediating roles of depression and family health
Source: BMC Psychiatry. 2025 Sep 26;25:875. doi: 10.1186/s12888-025-07353-7 (PMC12465402; doi:10.1186/s12888-025-07353-7)
Supplement: Supplementary file 2 — Supplementary Material 2. [file 12888_2025_7353_MOESM2_ESM.docx]

**Survey on Family Function, Mental Health, and Quality of Life of Primary and Secondary School Students During the Epidemic**

**Informed Consent Form**

Dear Student,

The epidemic has changed our living and learning habits. You are now invited to participate in a research and teaching activity. This activity is conducted by the medical staff of the Second Affiliated Hospital of Xi'an Jiaotong University, aiming to understand the family function, mental health, and quality of life of primary and secondary school students during the epidemic. The questionnaire consists of five parts and will take 10 to 15 minutes to complete. The survey is conducted on the principles of confidentiality and voluntariness. The research data will only be used for academic research purposes. The research results will not disclose any of your information when published, nor will it have any adverse effects on you personally. You will not receive any direct benefits from participating in this study, but your opinions on the survey content will help us propose suggestions for psychological interventions for primary and secondary school students. If you can complete this questionnaire survey in your spare time while studying, we sincerely thank you.

If you have any questions, you can contact the person in charge at any time: Yang Hui, Chief Nurse, Second Affiliated Hospital of Xi'an Jiaotong University, Phone: 13669253252, Email: yanghui.1226@163.com

Below are two options. Selecting option ① will take you to the questionnaire survey page; selecting option ② will automatically exit this page.

① I have read and understood the above information and voluntarily participate in this research.

② I do not agree to participate in this research.
