# Supplementary material for: A study on the relationship between learning burnout and quality of life among primary and secondary school students during an infectious disease epidemic: the mediating roles of depression and family health
Source: BMC Psychiatry. 2025 Sep 26;25:875. doi: 10.1186/s12888-025-07353-7 (PMC12465402; doi:10.1186/s12888-025-07353-7)
Supplement: Supplementary file 3 — Supplementary Material 3. [file 12888_2025_7353_MOESM3_ESM.docx]

**Survey on Family Resilience, Psychological Well-being, and Quality of Life of Primary and Secondary School Students in the Context of the Epidemic**

Survey Number: 9

IP Address: 1.83.246.60 (Shaanxi - Xi'an)

Source Channel: WeChat

Completion Time: 2022/1/19 14:45:10

Total Score: 299 (Average Score: 3.15)

*Informed Consent:

I have read and understand the information above and voluntarily participate in this study.

**Part One: Basic Information**

* A1. You are located in Xi'an, Shaanxi Province [Fill-in-the-blank question]

* A2. Your gender:

Female

* A3. Your age (years):

9

* A4. Your ethnicity:

Han

* A5. Current education stage:

Primary School

* A6. Your place of residence:

Urban

* A7. Epidemic prevention and control situation in your residence:

Control area

* A8. Your method of isolation:

Home isolation

* A9. Teaching method of your teacher:

Online teaching

**Part Two: Family Health Scale**

Instructions: Please indicate the extent to which you agree or disagree with the following statements describing your family. Answer these questions based on what you think about your family.

* B1. In my family, we support each other [Scale question]

Strongly agree (Score 5)

* B2. In my family, I feel secure in family relationships [Scale question]

Strongly agree (Score 5)

* B3. In my family, we help each other seek medical services when needed (e.g., registration) [Scale question]

Strongly agree (Score 5)

* B4. In my family, we help each other make changes for health [Scale question]

Strongly agree (Score 5)

* B5. In my family, we maintain hope even in very difficult times [Scale question]

Strongly agree (Score 5)

* ☆B6. In my family, we do not trust medical staff [Scale question]

Strongly disagree (Score 5)

* B7. When we encounter problems at school or work, our family can seek help from people outside the family [Scale question]

Strongly agree (Score 5)

* B8. If we need financial help, our family can borrow money from people outside the family (e.g., 1000 yuan) [Scale question]

Strongly agree (Score 5)

* ☆B9. In the past 12 months, after covering basic living expenses, our family has no spare money [Scale question]

Strongly disagree (Score 5)

* ☆B10. In the past 12 months, our family's housing cannot meet the needs of the family [Scale question]

Strongly disagree (Score 5)

****Part Three: Mental Health Questionnaire****

Instructions: Below are some situations or thoughts you may have had. Please choose the appropriate option according to your actual situation or feelings in the past week.

* None or almost none: In the past week, such situations did not occur for more than one day.

* A little: In the past week, such situations occurred for 1-2 days.

* Often: In the past week, such situations occurred for 3-4 days.

* Almost always: In the past week, such situations occurred for 5-7 days.

* C1. I am troubled by trivial matters [Scale question]

None or almost none (Score 3)

* C2. I don't feel like eating, my appetite is poor [Scale question]

None or almost none (Score 3)

* C3. Even with the help of family and friends, I still can't get rid of my inner distress [Scale question]

None or almost none (Score 3)

* ☆C4. I feel as good as most people [Scale question]

Almost always (Score 3)

* C5. When I am doing things, I cannot concentrate [Scale question]

None or almost none (Score 3)

* C6. I feel depressed [Scale question]

None or almost none (Score 3)

* C7. I feel that doing anything is very strenuous [Scale question]

None or almost none (Score 3)

* ☆C8. I feel that the future is hopeful [Scale question]

Almost always (Score 3)

* C9. I feel that my life is a failure [Scale question]

None or almost none (Score 3)

* C10. I feel afraid [Scale question]

None or almost none (Score 3)

* C11. My sleep is not good [Scale question]

None or almost none (Score 3)

* ☆C12. I feel happy [Scale question]

Almost always (Score 3)

* C13. I speak less than usual [Scale question]

None or almost none (Score 3)

* C14. I feel lonely [Scale question]

None or almost none (Score 3)

* C15. I feel that people are not very friendly to me [Scale question]

None or almost none (Score 3)

* ☆C16. I find life interesting [Scale question]

Almost always (Score 3)

* C17. I have cried [Scale question]

Almost always (Score 0)

* C18. I feel sad [Scale question]

None or almost none (Score 3)

* C19. I feel that people do not like me [Scale question]

None or almost none (Score 3)

* C20. I feel that I cannot continue my daily studies [Scale question]

None or almost none (Score 3)

****Part Four: Learning Burnout Scale****

Instructions: There is no right or wrong answer to all questions. We look forward to your true answers.

* ☆D1. I can devote myself to studying with energy. [Scale question]

Very consistent (Score 1)

* D2. Recently, I feel empty at heart and don't know what to do [Scale question]

Very inconsistent (Score 1)

* D3. My study is too poor, I really want to give up [Scale question]

Very inconsistent (Score 1)

* ☆D4. I can often achieve my goals [Scale question]

Very consistent (Score 1)

* D5. At the end of the day's study, I feel extremely tired [Scale question]

Not very consistent (Score 2)

* D6. I think I don't understand anyway, it doesn't matter whether I study or not [Scale question]

Very inconsistent (Score 1)

* ☆D7. When studying, I forget everything around me [Scale question]

Somewhat consistent (Score 2)

* D8. Recently, I often feel exhausted [Scale question]

Very inconsistent (Score 1)

* D9. In terms of learning, I don't feel a sense of achievement [Scale question]

Very inconsistent (Score 1)

* D10. I feel that learning is meaningless to me [Scale question]

Very inconsistent (Score 1)

* D11. I can handle exams well [Scale question]

Very consistent (Score 5)

* D12. At school, I often feel exhausted [Scale question]

Very inconsistent (Score 1)

* D13. I study with a cynical attitude [Scale question]

Very inconsistent (Score 1)

* ☆D14. I can effectively solve the problems that arise in my studies [Scale question]

Very consistent (Score 1)

* ☆D15. I can always easily deal with learning issues [Scale question]

Very consistent (Score 1)

* ☆D16. I can easily master the knowledge I have learned [Scale question]

Very consistent (Score 1)

****Part Five: Quality of Life Scale****

Instructions: This questionnaire is to understand your living conditions. It has nothing to do with whether you are smart or a good student, and there is no right or wrong answer to the questions. Please choose the answer that is closest to your feelings and life according to your actual situation.

* E1. Do you think your classmates are friendly to you? [Scale question]

Quite friendly (Score 3)

* E2. Can you easily participate in track and field and ball games? [Scale question]

Relatively easy but poor physical strength (Score 2)

* E3. Are you satisfied with your memory? [Scale question]

Very satisfied (Score 4)

* E4. Is there a place near your home where you can engage in physical activities? [Scale question]

There is a very good place (Score 4)

* E5. Are you satisfied with your relationship with teachers? [Scale question]

Very satisfied (Score 4)

* E6. Are you satisfied with your relationship with your parents? [Scale question]

Very satisfied (Score 4)

* E7. Do you have the opportunity to participate in extracurricular activities you like? [Scale question]

There are many opportunities (Score 4)

* E8. Do your friends care about you? [Scale question]

Very much care (Score 4)

* E9. Do you have many good friends? [Scale question]

Many (Score 4)

* E10. Are you satisfied with your ability to participate in sports activities? [Scale question]

Quite satisfied (Score 3)

* E11. Is the environment around your home quiet? [Scale question]

Very quiet (Score 4)

* E12. Are you satisfied with your sleep? [Scale question]

Very satisfied (Score 4)

E13. Are you satisfied with your energy? [Scale question] Very satisfied (Score 4)

E14. Are you satisfied with your life? [Scale question] Very satisfied (Score 4)

E15. Can you conveniently buy your living and learning supplies near your home? [Scale question] Convenient (Score 3)

E16. Do you think teachers like you? [Scale question] Very much like (Score 4)

E17. Are you satisfied with your health? [Scale question] Very satisfied (Score 4)

E18. Is the transportation near your home convenient? [Scale question] Very convenient (Score 4)

E19. Are you satisfied with your friends? [Scale question] Very satisfied (Score 4)

E20. Do you think your life is happy? [Scale question] Very happy (Score 4)

☆E21. Do you often regret what you have done? [Scale question] Always (Score 1)

E22. Do you often feel that most people like you? [Scale question] Never (Score 1)

☆E23. Do you feel annoyed as soon as you start your homework? [Scale question] Never (Score 4)

☆E24. Do you often need to take several breaks during your homework? [Scale question] Never (Score 4)

E25. When encountering difficulties, can you persist? [Scale question] Always (Score 4)

E26. When encountering difficulties, can you get help from teachers? [Scale question] Always (Score 4)

☆E27. Do you often prefer certain foods or not eat certain foods? [Scale question] Never (Score 4)

☆E28. Do you easily feel nervous or scared? [Scale question] Never (Score 4)

E29. When in need of help, can you find a trustworthy friend? [Scale question] Always (Score 4)

E30. Do you often easily remember new knowledge? [Scale question] Always (Score 4)

☆E31. Do you often feel pain or physical discomfort? [Scale question] Rarely (Score 3)

☆E32. Do you often feel troubled by this or that? [Scale question] Rarely (Score 3)

E33. Do you often have the opportunity to visit exhibitions, competitions, or travel? [Scale question] Rarely (Score 2)

E34. Besides completing homework, are you willing to do other exercises? [Scale question] Always (Score 4)

☆E35. Do you often worry about making mistakes? [Scale question] Never (Score 4)

E36. Do you feel important in the group? [Scale question] Never (Score 1)

E37. Do you often like to stay with your parents? [Scale question] Always (Score 4)

E38. Do you often raise your hand to answer questions posed by teachers? [Scale question] Always (Score 4)

☆E39. Do you often feel tired or lack energy? [Scale question] Never (Score 4)

☆E40. Do you often feel tired after getting up in the morning? [Scale question] Never (Score 4)

E41. Do you like your teachers? [Scale question] Always (Score 4)

E42. Do you think you are a good student? [Scale question] Rarely (Score 2)

☆E43. Do you often feel that life is meaningless? [Scale question] Never (Score 4)

E44. Can parents understand your thoughts? [Scale question] Rarely (Score 2)

☆E45. Do you often take a long time to complete your homework? [Scale question] Never (Score 4)

E46. Do you think teachers are friendly to you? [Scale question] Always (Score 4)

☆E47. Do you often feel like not eating or overeating? [Scale question] Never (Score 4)

E48. Do you often participate in physical exercises? [Scale question] Often (Score 3)

E49. When encountering difficulties, are you willing to tell your parents? [Scale question] Never (Score 1)

Thank you again for your participation!
